# Archaeolinguistic evidence for the farming/language dispersal of Koreanic

**DOI:** 10.1017/ehs.2020.49

**Published:** 2020-10-14

**Authors:** Mark J. Hudson, Martine Robbeets

**Affiliations:** Eurasia3angle Research Group, Department of Archaeology, Max Planck Institute for the Science of Human History, Jena 07745, Germany

**Keywords:** Archaeolinguistics, Neolithic, agriculture, Korea, Transeurasian, *Yersinia pestis*

## Abstract

While earlier research often saw Altaic as an exception to the farming/language dispersal hypothesis, recent work on millet cultivation in northeast China has led to the proposal that the West Liao basin was the Neolithic homeland of a Transeurasian language family. Here, we examine the archaeolinguistic evidence used to associate millet farming dispersals with Proto-Macro-Koreanic, analysing the identification of population movements in the archaeological record, the role of small-scale cultivation in language dispersals, and Middle–Late Neolithic demography. We conclude that the archaeological evidence is consistent with the arrival and spread of Proto-Macro-Koreanic on the peninsula in association with millet cultivation in the Middle Neolithic. This dispersal of Proto-Macro-Koreanic occurred before an apparent population crash after 3000 BC, which can probably be linked with a Late Neolithic decline affecting many regions across northern Eurasia. We suggest plague (*Yersinia pestis*) as one possible cause of an apparently simultaneous population decline in Korea and Japan.

## Graphical abstract

**Figure:**
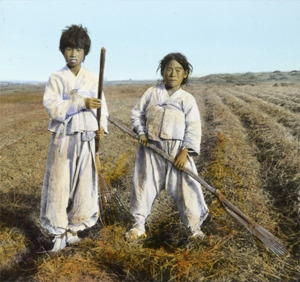

**Media summary**: Archaeolinguistics supports the ancestor of the Korean language reaching the Korean peninsula in association with millet farming in the Neolithic. A population decrease on the Korean peninsula after around 3000 BC appears to be part of a broader Late Neolithic decline recognized in many areas of Eurasia. Plague (*Yersinia pestis*) may have been one cause of this decline in Korea and Japan.

## Introduction

The farming/language dispersal hypothesis proposes that demographic growth amongst early farmers led to population expansions from homeland regions. Linguistic change occurred not only through the geographical expansion of the languages of farmers but also by hunter–gatherer language shift (Bellwood & Renfrew, 2002; Diamond & Bellwood, 2003). Based originally on analyses of Austronesian and Indo-European (Renfrew, 1987; Bellwood, 1991), evidence supporting farming/language dispersals has long been discussed for many other regions (e.g. Phillipson, 1997; Diakonoff, 1998; Glover & Higham, 1996; Bellwood & Renfrew, 2002). Although Japonic has also been linked with a farming dispersal (Hudson, 1994, 1999; Lee & Hasegawa, 2011; Whitman, 2011), some scholars have noted that pastoralism and processes of elite dominance make the farming/language dispersal hypothesis difficult to apply to the north Eurasian languages classified as Altaic or – *sensu* Johanson and Robbeets (2010) – as Transeurasian (e.g. Renfrew, 1992, pp. 30–32; Heggarty & Beresford-Jones, 2014).

Recently, however, archaeobotanical research identifying northeast China as a centre of millet domestication has enabled linguists to propose the West Liao basin as the Neolithic homeland of a Transeurasian language family (Robbeets, 2017a, b, 2020). As part of this new work, the farming/language dispersal hypothesis has been systematically applied to the Korean peninsula for the first time through the suggestion that Proto-Macro-Koreanic arrived with millet cultivation around 3500 BC (Robbeets, 2017a, b, Robbeets et al., 2020). Here, we provide a new analysis of the archaeological evidence used to associate millet farming dispersals with Proto-Macro-Koreanic before discussing linguistic data which support an early arrival of Proto-Macro-Koreanic on the peninsula.

## Korean archaeology and ethno-linguistic origins: background

The farming/language dispersal hypothesis in Korea needs to be first placed in the context of broader discourse over the evolution of human society on the peninsula. Owing largely to the legacy of Japanese colonialism, migration and ethnicity have been controversial topics in Korean history and archaeology (Pai, 1994, 1999, 2000; Nanta, 2007; Kim, 2008; Park & Wee, 2016). During Japan's colonial rule (1910–1945), the ‘backwardness’ of Korean civilization was emphasized, as was the insistence that historical change had derived from outside influence. Colonial interpretations stressed the racial and cultural ‘inferiority’ of the Korean people and their dependence on outside stimuli (Kim, 2008, p. 124). At the same time, using linguistic as well as archaeological evidence, colonial scholars expounded the theory that the Korean and Japanese peoples shared a common ancestor in prehistory – the so-called *Nissen dōsoron* (Oguma, 2002, pp. 64–92). Research on Korean origins following independence has to be understood as a critique of this colonial discourse.

In the 1960s, archaeologist Jŏng-hak Kim made the influential argument that the Korean people were formed in the Bronze Age through the replacement of a Neolithic ‘Palaeo-Siberian’ (or ‘Palaeo-Asiatic’) population by an Altaic Tungusic-speaking tribe known as the Yemaek. This thesis became widely adopted in Korean archaeology (Park & Wee, 2016, p. 313; Pai, 2000, pp. 79–80) and, in fact, in Korean society as a whole (Hong, 2006, p. 23). As a result, it has been assumed that while the introduction of rice cultivation in the Bronze Age marked a major transition, the earlier cultivation of millets had resulted from small-scale cultural diffusion from northeast China (Kim, 1986). The Neolithic was to be understood as the time of ‘indigenous Korean hunter–fisher–gatherers’ (Shin et al., 2013, p. 69). As an extension of this perspective, research on the adoption of farming has often emphasized regional and chronological variation across the peninsula (Kwak & Marwick 2015; Bale, 2017; Kwak, 2017; Kwak et al., 2017; Kim et al., 2018).

The periodization and chronology used in the present paper are shown in Table 1. The term ‘Chulmun’ (also romanized as Jeulmun) is sometimes applied to the Neolithic as a whole but more properly refers to the Middle and Late phases. Chulmun or ‘comb-pattern’ was coined in 1930 by Japanese colonial archaeologist Ryōsaku Fujita on the basis of what he saw as similarities between Korean pottery and the *Kammkeramik* of northern Eurasia (Kim, 1978, p. 10). More recently, other styles of Neolithic pottery have also been identified. Currently, the earliest Korean ceramics are from Cheju island with reported dates as early as 9920 BC, although this pottery tradition is said to have flourished especially after 7600 BC (G.K. Lee et al., 2019; Shoda et al., 2017). Appliqué *yunggimun* pottery, which may have been influenced by the Amur region, appears on the east coast of the peninsula in the sixth millennium BC (Shin et al., 2012). Chulmun pottery associated with sedentary villages appears around 4000 BC or slightly earlier (Shin et al., 2012). There is still no consensus on the origins of the Chulmun ceramic tradition, although links with Liaodong have been suggested (Xu, 1995).

**Table 1.** Korean archaeological chronology for the Neolithic and Bronze Ages. Based on Bausch (2016) with modifications.

Period	Dates BC	Representative ceramic styles
Incipient Neolithic	7500–5000	Konamri plant-tempered pottery
Early Neolithic	5000–3500	Appliqué *yunggimun* pottery
Middle Neolithic	3500–2000	Chulmun comb-pattern pottery
Late Neolithic	2000–1300	Chulmun comb-pattern pottery
Bronze Age	1300–400	Mumun plain pottery

Hunter–gathering was a major element of the subsistence economy of Neolithic Korea. The practice of some form of Neolithic agriculture had long been raised as a possibility (Nelson, 1993), but new finds are continuing to transform the field (Bale, 2001; Lee et al., 2019; Kwak et al., 2020). *Chenopodium* sp., soybeans (*Glycine max*) and adzuki beans (*Vigna angularis*) may already have been grown by the Early Neolithic (Shin et al., 2012, p. 76; Lee et al., 2019; Kwak et al., 2020). Broomcorn (*Panicum miliaceum*) and foxtail (*Setaria italica*) millets reached Korea by the fourth millennium BC (Lee, 2017a, b). Following this, a new agricultural system with millets, rice (*Oryza sativa*), barley (*Hordeum vulgare*) and wheat (*Triticum aestivum*) developed in Korea by 1300 BC (Ahn, 2010; Kwak et al., 2017; Kim et al., 2019; Lee, 2011, 2017a, b). Although Japanese archaeologists had earlier suggested that rice had been introduced to Korea *from* Japan, by the 1970s it was accepted that rice had moved to the peninsula from northeast China (Kim, 1982) and that Bronze Age agriculture spread from Korea *to* Japan after 1000 BC (Crawford, 2018; de Boer et al., 2020; Li et al., 2020; Miyamoto, 2014, 2016; Leipe et al., 2020).

Working out a linguistic chronology for the Korean language is more challenging, because the written records for Korean are relatively late and, unlike archaeological evidence, linguistic evidence cannot be excavated from the ground. To explore the history of the language before it has been actually written down, linguists rely upon the comparison of Korean with other languages. In addition to identifying early borrowings, this allows them to infer unattested ancestral states of Korean, referred to as ‘proto-languages’, meaning the common source of all of the languages in a given family.

**Figure 1.**
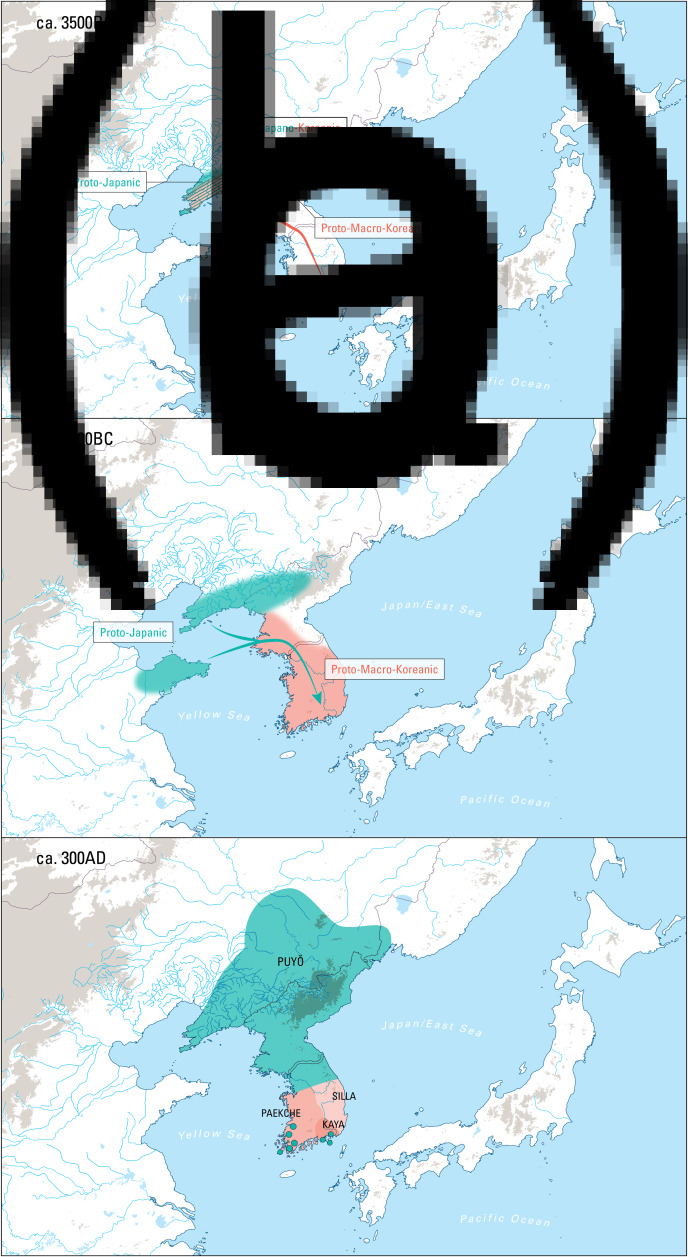
The linguistic landscape of the Korean peninsula in time and space. 1a: ca. 3500 BC Proto-Macro-Koreanic separates from Proto-Japanic on the Liaodong peninsula and enters the Korean peninsula; 1b: ca. 1500 BC Proto-Japanic enters the Korean peninsula from the Liaodong and Shandong peninsulas; 1c: ca. 300 AD Japanic Puyŏ languages are spread from the Liaodong peninsula to the Korean peninsula; the Macro-Koreanic Han languages, Paekche, Kaya and Silla are situated in the south of the Korean peninsula; pockets of Japanic languages are scattered among Paekche and Kaya languages.

Three genealogical hypotheses about the origins of Korean are taken seriously today: the Transeurasian hypothesis (Ramstedt, 1949; Lee, 1977; Martin, 1996; Miller, 1996; Starostin et al., 2003; Robbeets, 2005, 2015), the hypothesis that only Korean and Japanese are related (Martin, 1966, 1991; Whitman, 1985, 2012; Unger, 2009; Francis-Ratte, 2016), and the possibility that Korean is an isolated language like Ainu or Nivkh, without living relatives today (Janhunen, 2010; Vovin, 2005). Proto-Transeurasian is the name of the ancestral language from which the Turkic, Mongolic, Tungusic, Japanic and Koreanic languages are thought to descend, while Proto-Japano-Koreanic is a daughter of Proto-Transeurasian to which both the Japanic and Koreanic languages can be traced back. Before the separation of the two language families around 3500 BC, Proto-Japano-Koreanic was probably spoken along the Bohai coast and on the Liaodong peninsula (Unger, 2014; Francis-Ratte, 2016; Robbeets, 2020; see Figure 1a). The term ‘Proto-Japanic’ refers to the ancestor of the historical continental varieties of the Japanese language as well as the varieties spoken on the Japanese Islands, while the label ‘Proto-Japonic’ is restricted to the branch of Japanic that is ancestral to Mainland Japanese and the Ryukyuan languages.

We do not have any historical information about the languages spoken on the Korean peninsula until the third century AD, when Chinese dynastic chronicles start to leave some vague records of languages spoken at that time and how they related to each other. According to these sources, the local inhabitants were roughly divided into three ethno-linguistic groups: the Sushen, the Puyŏ and the Han. The Sushen people consisted of northern semi-nomadic tribes and are usually associated with the ancestors of the Jurchen, who spoke a Tungusic language (Janhunen, 1996; Beckwith, 2004). The Puyŏ were scattered over the Liaodong peninsula and the northern half of the Korean peninsula and included four groups, the Puyŏ proper, Koguryŏ, Okchŏ and Ye. Their language was probably more closely related to Japanese than to Korean (Beckwith, 2004, 2005). The Han – who are not to be confused with the Chinese Han – consisted of three related groups of people in the southern part of the Korean peninsula. In the Three Kingdoms period (AD 300–668), the Mahan in the west became Paekche, the Pyŏnhan in the Naktong River valley in the centre became Kaya, and the Chinhan in the east became Silla, each with their individual languages (Figure 1c). The Han languages are usually associated with various Koreanic languages (Lee and Ramsey, 2011); their ancestor can be specified as ‘Proto-Macro-Koreanic’, even if many linguists use ‘Proto-Koreanic’ as a general term to refer to the entire period between the separation from Proto-Japanic and the break-up into the predecessors of contemporary varieties.

Nevertheless, as indicated by the green dots in Figure 1c, there must have been pockets of Japanic speech communities among the Koreanic languages. Chinese chronicles, such as the *Hou Han Shu* (the fifth century AD ‘History of the Later Han’), state that the Pyŏnhan people were close to the Wa, the ethnonym for inhabitants of the Japanese islands. Linguistically, the alleged presence of Japanic languages in Korea is confirmed by the historical Japanic toponyms, documented especially in the Mahan and Pyŏnhan regions (Bentley, 2000) and by a small number of words in the *Nihon shoki* which might be of Kaya origin (Kōno, 1987). Therefore, it seems likely that there were at least some Japanic languages among the Pyŏnhan and Mahan languages.

However, the Silla kingdom unified the Korean peninsula politically and linguistically in 668, erasing all pre-existing linguistic diversity. Owing to this unification, the contemporary Korean dialects cannot be traced back any deeper in time than the end of the first millennium AD. Thus, the separation of Proto-Koreanic into the predecessors of the contemporary dialects corresponds roughly to the time of the break-up of Silla Old Korean. Thus, similar to the distinction between Latin and Proto-Romance, Silla Old Korean is an – albeit very fragmentary – historically attested language, while Proto-Koreanic is the language reconstructed through the comparison of contemporary and historically well-attested varieties of Korean.

**Figure 2.**
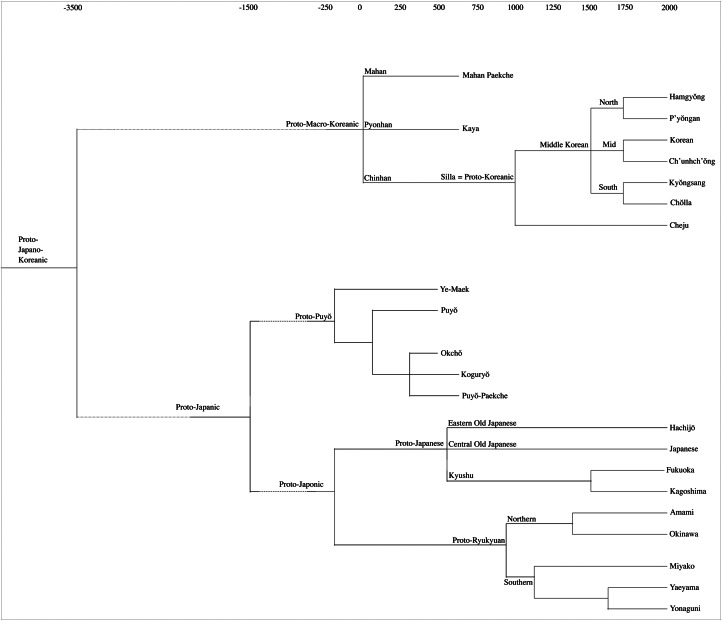
Classification of Japano-Koreanic based on classical comparative historical linguistic inferences (adapted from Robbeets, 2015).

It is believed that the language of Silla was the direct ancestor of Middle and Contemporary Korean. Since the Old Silla and Old Paekche Korean material mainly consists of individual words and a few poems, and since the exact phonological value underlying the Chinese characters of Early Middle Korean records is unclear, it is not until Late Middle Korean, the language written down after the invention of the Korean script in 1446, that we get a thorough linguistic understanding of the Korean language. The linguistic periodization and chronology used in the present paper are shown in Table 2, while a classification of Japano-Koreanic is proposed in Figure 2.

**Table 2.** Korean linguistic chronology

Language variety	Dates
Proto-Transeurasian	Before 4700 BC
Proto-Japano-Koreanic	Ca. 4700–3500 BC
Proto-Macro-Koreanic	Ca. 3500 BC to AD 300
Proto-Koreanic	AD 300–1000
Silla Old Korean	AD 300–918
Paekche Old Korean	AD 300–668
Kaya Old Korean	AD 300–668
Early Middle Korean	AD 918–1446
Late Middle Korean	AD 1446–1592

## Koreanic and the farming/language dispersal hypothesis

Three common assumptions regarding the spread of millet farming in Neolithic Korea are relevant to the farming/language dispersal hypothesis. These assumptions are that millet cultivation was (a) not associated with major transformations in material culture or settlement patterns, (b) did not affect the continued primary importance of wild plants and marine resources and (c) did not lead to population increase. These observations are contrasted with the Korean Bronze Age (1300–400 BC), which is widely argued to be characterized by abrupt shifts in material culture, the replacement of hunter–gathering by rice farming, and by clear evidence for demographic growth. In the following, these arguments are discussed in turn.

*Did millet farming spread to Korea through migration or diffusion?*

Some archaeologists have argued that the archaeological record supports immigration into the Korean peninsula in the Bronze Age but not in association with the introduction of millet farming in the Neolithic (Ahn, 2010; Kim & Park, 2020). In this section we argue that evidence relating to sedentism, pottery, stone tools and weaving technology is, in fact, consistent with population movements into Korea in the fourth millennium BC.

Migrations have always been a controversial problem in archaeology owing to the methodological difficulties of identifying population movements from the archaeological record, as well as theoretical issues over the role of migration and ethnicity in historical change (Bellwood, 2013; Burmeister, 2017). Traditions of historiographic research also influence how scholars are predisposed to view continuity vs. discontinuity in the archaeological record. The relationship between material culture and ethnic and linguistic identity has been seen as a key issue. In archaeology, classical methods, such as those used by Rouse (1986), attempt to link migrations with more or less sudden changes in archaeological sequences. Where evidence from biological anthropology and linguistics can be combined with archaeology, then a convincing argument can often be made for migration (Hudson, 1999). The difficulty is how to interpret cases without sudden breaks in the archaeological record or without biological evidence.

Ethnographic work by Leach (1954), Barth (1969) and others transformed the way we see ethnicity. No longer a fixed or ‘primordial’ identity, such research demonstrated that ethnicity is often used in a contextual or strategic fashion. Hodder (1982) began the study of the implications of this understanding for material culture. Against this background, the farming/language dispersal hypothesis marked an important theoretical contribution to the archaeology of population movements in attempting to organize the previously rather undisciplined research on archaeology and language dispersals (Renfrew, 1992). New work using ancient DNA has further transformed research, leaving little doubt that population movements did play an important role in Neolithic and later transitions to farming (Haak et al., 2010; Isern et al., 2017; Gamba et al., 2012; Mathieson et al., 2015; Racimo et al., 2019; Shennan, 2018).

In Korea, the Middle Neolithic of the fourth millennium BC saw increasing sedentism, a process which seems to have spread from the central-west to the southern peninsula. Ahn et al. (2015, p. 113) note that ‘Small-scale millet cultivation introduced from northern China seems to have been adopted by Neolithic hunter–gatherers almost simultaneously’ with this shift to sedentism. From the latter part of the Middle Neolithic, the presence of millet farming villages along rivers in the southern peninsula and on the eastern and southern coasts is noted by Shin et al. (2012). Although Shin and colleagues see these as small, shifting settlements, Korean archaeologists such as Eun-Sook Song have argued that the expansion of Middle Neolithic culture across the southern part of the peninsula was a farming dispersal (discussed in Shin et al., 2012, p. 83). Millet cultivation and sedentism were associated with Chulmun comb-pattern ceramics, which also spread from the central-west zone across the southern peninsula (Ahn et al., 2015; Lee, 2017a). The average size (volume) of pottery increases during this phase, suggesting an important role in the storage of food. Ahn et al. (2015, p. 114) argue that the Chulmun culture ‘adopted small-scale millet cultivation through interacting with people in the Liaoning area in China’, but population movement is an equally reasonable explanation given that, as discussed below, other aspects of material culture also change at this time.

From west-central Korea, millet farming spread to the southern peninsula, not just with Chulmun pottery but in association with a range of stone tools such as grinding stones, pestles, sickles, waisted hoes, digging sticks and polished stone arrowheads (Choe & Bale, 2002; Shin et al., 2012; Miyamoto, 2014; Ahn et al., 2015). With the exception of stone arrowheads, these are all tools that could reasonably be linked with agriculture, although the processing of nuts is of course another possibility for the grinding stones. As discussed in detail by Nelson et al. (2020), textile technology provides further support for Neolithic farming dispersals in Northeast Asia, including Korea. Textile weaving, often using spindle whorls, became widespread in temperate climatic zones from the early Holocene (Gilligan, 2019). The oldest spindle whorls in Korea have been reported from a few Early Neolithic sites possibly related to incipient millet cultivation (Nelson et al., 2020, pp. 11, 14). Yet an increase in the number and stylistic similarities of whorl finds from the Middle Neolithic is consistent with the linguistic evidence for a farming/language dispersal of Proto-Macro-Koreanic in the fourth millennium BC (Nelson et al., 2020).

The archaeological evidence summarized above does not constitute undeniable proof that a migration from northeast China into Korea occurred during the fourth millennium BC. The evidence is, however, broadly consistent with such an interpretation and with the expectations of the farming/language dispersal hypothesis.

*How important was millet farming in Middle–Late Neolithic subsistence in Korea?*

Many archaeologists have argued that Neolithic millet cultivation in Korea was an addition to a hunter–gathering economy but did not mark a major subsistence transformation. While some scholars have advised caution in light of the relative scarcity of archaeobotanical analyses (Crawford & Lee, 2003), this interpretation of the Neolithic economy is common (Bae et al., 2013; Kim, 2010, 2014; Kim & Park, 2020; Shin et al., 2012). However, the farming/language dispersal hypothesis does not require that farming form the only component of a subsistence economy. As discussed in the next section, even small-scale cultivation of millet might have been associated with language dispersal and shift, a conclusion which also appears warranted for the Primorye province of the Russian Far East (Li et al., 2020).

The Korean peninsula packs different landscapes, such as forests, grasslands, mountains and freshwater and maritime environments, into a rather small area, a diversity that probably helped to maintain resilience at times of climate change. The fourth millennium BC has been analysed as a period when a colder and drier climate began around 3500 –3300 BC (Ahn et al., 2015). While the exact chronology and impacts of such climate change will no doubt continue to be debated, adding millet agriculture to a variety of other subsistence strategies, including hunting, gathering and fishing, probably helped Chulmun populations expand into different environments and adapt to the changing climate. In Neolithic Northeast Asia, broad-spectrum subsistence was common in regions such as the West Liao basin and the southern Primorye, but this does not contradict the basic tenets of the farming/language dispersal hypothesis (Robbeets, 2017b; Li et al., 2020).

Isotope analyses have been used to argue that marine resources and wild plants, not millets, formed the main diet of the Chulmun people (Kim & Park, 2020, p. 8). However, many isotope studies are from coastal shell middens, where human bones are usually better preserved than in inland sites. Other research has identified regional variation in Neolithic isotope values from Korea (Choy et al., 2012). Neolithic shell midden sites on the Korean peninsula, especially those from the eastern and southern coasts, demonstrate the importance of marine resources, including the specialized capture of large species such as sea lions, sharks and whales. However, this specialist use of marine resources does not necessarily contradict the importance of millet farming. In Japan, for example, the transition to agriculture in the first millennium BC was marked by the increased importance of specialized fishing, which was perhaps oriented towards trade with farmers (Hudson, 2019a; Takase, 2020; cf. Ling et al., 2018).

*Why did population decline in the Late Neolithic?*

Recent studies of prehistoric demography on the Korean peninsula have been interpreted as evidence that population did not increase after the introduction of millet farming. The assumption here is that language shift requires a large initial influx of speakers; the possibility that Proto-Macro-Koreanic was introduced by a small number of speakers who later grew in number is not considered. The farming/language dispersal hypothesis proposes that agriculture enables populations to generate and store more food per area of land (Greenhill, 2015). Even if millet provided a relatively minor contribution to Neolithic diets in Korea, millet cultivation can still be expected to have increased the resilience of Neolithic populations, enabling them to obtain and store more food. Bettinger and Baumhoff (1982) demonstrated that even small technological advantages can impact language shift, a phenomenon which probably also explains the expansion of Pama–Nyungan languages across Australia (Evans & Jones, 1997; Bouckaert et al., 2018). The advantages accrued from millet cultivation in Neolithic Korea might have been sufficient for the spread of Proto-Macro-Koreanic.

**Figure 3.**
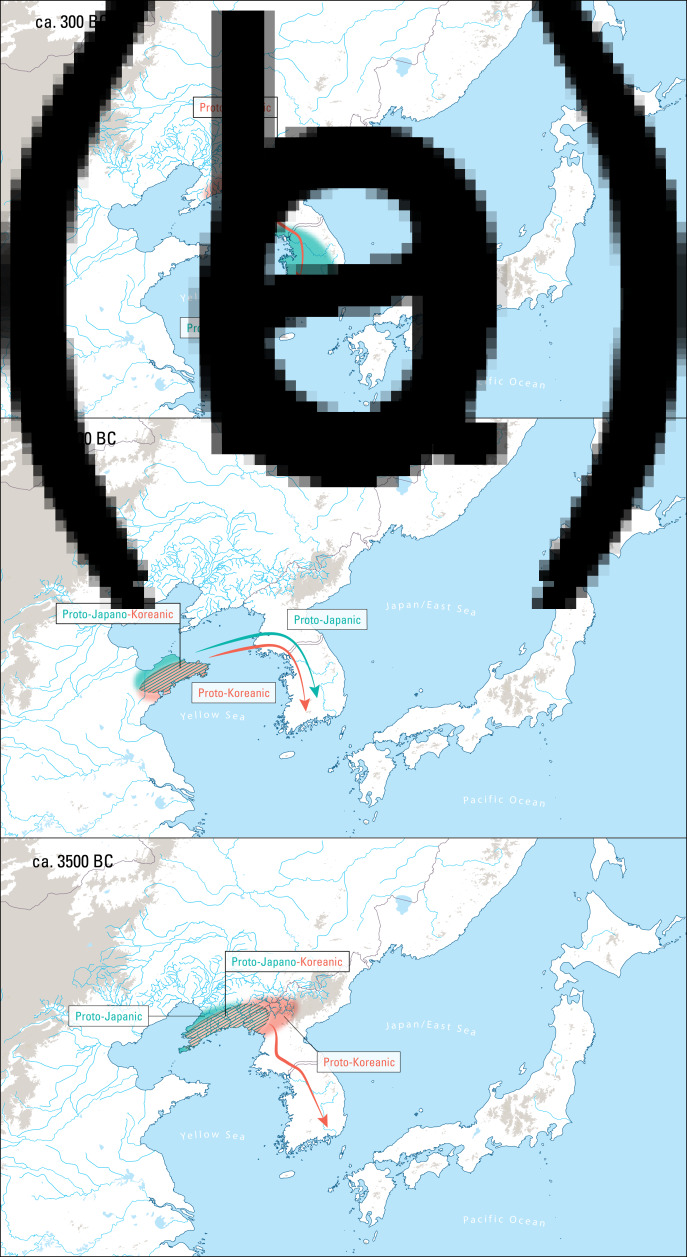
Different hypotheses for the arrival of Proto-Macro-Koreanic on the Korean peninsula. (a) Proto-Macro-Koreanic arrived after Proto-Japanic from Liaodong and the Changbaishan region with the introduction of bronze daggers around 300 BC (Whitman 2011). (b) Proto-Macro-Koreanic arrived simultaneously with Proto-Japanic from the Liaodong and Shandong peninsulas with the introduction of rice agriculture around 1300 BC (Kim & Park, 2020). (c) Proto-Macro-Koreanic arrived before Proto-Japanic from the Liaodong peninsula with the introduction of millet agriculture around 3500 BC (Robbeets, 2017a, b).

Neolithic millet cultivation in Northeast Asia probably spread through extensive, low-intensity land use (Stevens & Fuller, 2017; Qin & Fuller, 2019), an ecological expectation consistent with archaeological evidence from the Primorye (Li et al., 2020). Previous research in Korea has noted an ‘explosive’ increase in the number and size of Middle Neolithic settlements (Shin et al., 2012, pp. 85–87). Ahn et al. (2015, p. 135) write that, ‘After settlements with millet cultivation appeared in the early fourth millennium BC in central-western Korea, the population increased rapidly until c. 3500 cal BC. After that, settlements spread to other areas including the Geum [Kŭm] River valleys and central-east coast.’ Despite this earlier research, Kim and Park (2020) emphasize that a population decline after the introduction of millet is evidence that a large migration into the Korean peninsula did not occur in the fourth millennium BC. However, Kim and Park's figure 1, adapted from Ahn et al. (2015), places the arrival of millet at 3500 BC; if millet had reached Korea a century or two earlier – a possibility supported by Ahn et al. (2015) and G.A. Lee et al. (2019) – then figure 1 would show a significant initial *increase* in population with millet farming. Kim and Park's figure 3 is based on an earlier study concluding that the introduction of millet to Korea was not associated with rapid population growth and that population actually *declined* after 2900 BC (Oh et al., 2017). While, as noted above, high population levels were not necessarily required for a shift to Proto-Macro-Koreanic, the demographic data summarized by Kim and Park are important to understanding language dispersals in Northeast Asia.

Recently, archaeology has brought the discontinuous nature of crop dispersals in Northeast Asia into sharper focus (Leipe et al., 2019; Stevens & Fuller, 2017). Early millet expansions to Korea and the Primorye in the fourth millennium BC were followed by a period of more than 2500 years before farming spread from Korea to Kyushu, a distance of only 200 km. Another hiatus occurred before agriculture then spread to the lower Amur, Hokkaido and the Ryukyus from the late first to early second millennia AD (Crawford & Yoshizaki, 1987; Takamiya et al., 2015; Hudson, 2017; Leipe et al., 2017). Previous research has linked these later dispersals to population movements but has rarely attempted to understand periods of stasis. In Korea, although there seems to have been an increase in population in the fourth millennium BC – presumably linked to the arrival of millet cultivation – after around 3000 BC a population *decline* seems to be well supported (Ahn et al., 2015; Oh et al., 2017).

The resilience of early farming systems was often low, leading to ‘boom and bust’ cycles of both agriculture and language dispersal (Atkinson et al., 2008; Stevens & Fuller, 2012; Shennan et al., 2013; Colledge et al., 2019; Hudson, 2019b). A general Late Neolithic decline has been proposed for several regions of Eurasia, including Europe, China and Japan. Climate change, immigration of steppe pastoralists, trade and plague (*Yersinia pestis*) are possible causes of this decline (Kristiansen, 2015; Hosner et al., 2016; Rascovan et al., 2019). This decline was noticed very early in Japan where Koyama (1981) estimated that population levels in the third millennium BC dropped almost 40% across Kyushu, Shikoku and Honshu, and by almost 60% in central Honshu – rates comparable with the effects of the Black Death in Europe. Later research has supported the general trends identified by Koyama using site and pit house numbers as well as radiocarbon dates (Imamura, 1996, pp. 95–96; Hudson, 1999, p. 140; Yano, 2014; Crema et al., 2016; Crema & Kobayashi, 2020). The precise chronology is difficult but the most recent study concludes a starting date for the decline in the Kanto and Chubu regions of 4900 cal BP (Crema & Kobayashi, 2020).

Epidemic disease was suggested as a possible cause of the Late Neolithic decline in Japan by Oikawa and Koyama (1981) and Kidder (1995, 2007). Recent findings of *Y. pestis* from Neolithic Sweden in an individual dated 5040–4867 BP (Rascovan et al., 2019) and in two individuals dated to 4556 and 4430 BP from Lake Baikal (Yu et al., 2020) suggest the need to re-consider role of epidemic disease in the Late Neolithic decline in Northeast Asia, a point also made by Hosner et al. (2016). As in Japan, the period from the third millennium BC in Korea is associated with settlement decentralization, broader-spectrum resource use and a move towards greater maritime mobilities. While the role of plague in these changes awaits confirmation from biomolecular analyses, the broader context would seem to be increased Bronze Age connectivities across Eurasia (Hudson et al., in press).

## Linguistic evidence for the arrival of Macro-Koreanic: evaluating the hypotheses

Today most linguists (Janhunen, 2010; Unger, 2005; Whitman, 2011; Beckwith, 2005; Francis-Ratte, 2017; Robbeets et al., 2020; Kim & Park, 2020) would agree that at least two different language families, Macro-Koreanic and Japanic, coexisted on the Korean peninsula in the first millennia BC and AD. Although these scholars agree to associate the introduction of rice agriculture to the Korean peninsula around 1300 BCE with the dispersal of Proto-Japanic, there is disagreement about the timing and the spread model of Proto-Macro-Koreanic. Figure 3 illustrates the different hypotheses for the arrival of Proto-Macro-Koreanic on the Korean peninsula.

As shown in Figure 3c, some scholars proposed that Proto-Macro-Koreanic was spoken on the Korean peninsula from at least the second millennium BC, before the Bronze Age expansion to Japan in the Yayoi period (900 BC to AD 250; Janhunen, 1999, 2005), or before the advent of Proto-Japanic with Bronze Age Mumun culture (1300–400 BC; Vovin, 2005; Beckwith, 2004, 2005; Robbeets, 2017a, b, 2020). Others suggested that it post-dated Proto-Japanic and arrived on the Korean peninsula between the third century BC and the fourth century AD (Unger, 2005; Whitman, 2011; see Figure 3a). Among the supporters of an early arrival, Robbeets (2017a, b) proposed a concrete spread model, linking it to the adoption of millet agriculture, while among the supporters of a late arrival, Whitman (2011) associated it with the introduction of bronze daggers around 300 BC and Unger (2005) with the rise of the Silla kingdom in the fourth century AD.

Recently, Kim and Park (2020) have rejected both Robbeets’ and Whitman's dispersal hypotheses for Proto-Macro-Koreanic, proposing two alternative scenarios, namely (a) that Proto-Macro-Koreanic arrived simultaneously with Proto-Japanic at the beginning of the Mumun period around 1300 BC or (b) that Proto-Japano-Koreanic arrived around 1300 BC before it separated into Japanic and Macro-Koreanic branches. The first scenario is illustrated in Figure 3b.

Both of these hypotheses pose serious problems. The first scenario associates the incoming Mumun farmers with two different ethno-linguistic groups of Macro-Koreanic and Japanic speakers. First, as Kim and Park (2020, p. 14) themselves admit, this is difficult to reconcile with the observation that ‘Early Mumun material culture and technology were homogenous throughout the peninsula, making it difficult to distinguish the two groups.’

Second, the observation that the Puyŏ languages, spoken on the Liaodong peninsula and the northern half of the Korean peninsula by the beginning of the Eastern Han Dynasty (AD 25–220), were more closely related to Japanese than to Korean (Beckwith, 2004, 2005; Robbeets, 2007) suggests that the languages spoken in the Liaodong area after the separation of Proto-Japano-Koreanic were descendants of Japanic rather than of Macro-Koreanic. This implies that Japanic speakers remained in the Liaodong area, while Macro-Koreanic speakers moved and left the area after the separation of Proto-Japano-Koreanic in the fourth millennium BC (see Figure 1 and Table 2).

Third, there are a number of linguistic indications that the Macro-Koreanic speakers added rice technology to a pre-existing agricultural package through borrowing from Japanic speakers, who, in turn, borrowed rice terminology from their earlier continental neighbours in the Shandong area, including, among others, speakers of Para-Austronesian languages, i.e. sister languages of proto-Austronesian (Stevens & Fuller, 2017; Robbeets, 2017b). The repurposing of agricultural vocabulary as rice terminology in Korean, for instance, supports the idea that the speakers of Koreanic were already familiar with agriculture before adding rice to their agricultural package. For example, Proto-Koreanic *pap originally meant ‘any boiled cereal’ and Proto-Koreanic *pʌsal ‘hulled (of any grain); hulled corn of grain’, but later these meanings specified into ‘boiled rice’ and ‘hulled rice’, respectively (Francis-Ratte, 2017; Robbeets et al., 2020). The proposed borrowing of Proto-Koreanic terms such as *pye ‘(unhusked) rice’ and *pʌsal ‘hulled (of any grain); hulled corn of grain; hulled rice’ from Proto-Japanic *ip-i ‘cooked millet, steamed rice’ and *wasa-ra ‘early ripening (of any grain)’ (Vovin, 2016; Robbeets et al., 2020), and of Proto-Japanic terms such as *kəmai ‘dehusked rice’ and *usu ‘(rice and grain) mortar’ from Para-Austronesian *Semay ‘cooked rice’ and *lusuŋ ‘(rice) mortar’ (Sagart, 2011; Robbeets, 2017b) is indicative of the direction of the diffusion, namely from Para-Austronesian into Japanic in the Shandong-Liaodong interaction sphere and, subsequently, from Japanic into Koreanic on the Korean peninsula.

And finally, there are traces of a pre-Nivkh substratum in Koreanic and of a pre-Ainu substratum in Japonic, but there is no evidence for Nivkh underlying features in Japonic that are unique in the sense that they are not characteristic for Ainu as well. Ainu and Nivkh represent marginal pockets of earlier languages whose lineages became isolated after the large-scale farming/language dispersals of various Transeurasian languages. Geography and linguistic distribution led to the assumption that at least some groups of hunter–gatherers on the Korean peninsula spoke ancestral varieties of Nivkh, while at least some hunter–gatherers on the Japanese islands spoke ancestral varieties of Ainu before the advent of agriculture. If the speakers of Japanic had arrived on the Korean peninsula prior to the speakers of Koreanic, we would expect more substratum interference from Nivkh in Japanic than in Koreanic. In reality, the opposite is true: we find indications of a pre-Nivkh substratum in Koreanic, while there are traces of a pre-Ainu substratum in Japonic, but even if there are proto-typical features that distinguish Nivkh from Ainu underlying in Koreanic, there are none in Japonic. For instance, the development of initial consonant clusters and three laryngeal contrast sets for stops in Koreanic may be due to substratum influence from pre-Nivkh, but these features are absent from Ainu as well as from Japonic. In contrast, features such as the occurrence of prefixing morphology in spite of the verb–final word order and the distinction between intentional and non-intentional action in certain verb affixes in Japonic may be due to substratum influence from pre-Ainu, but these features are absent from Nivkh as well as from Koreanic. These observations suggest that Macro-Koreanic – not Japanic – was the first language adopted by early hunter–gatherers on the Korean peninsula.

Kim and Park's second hypothesis, namely that the ancestral Proto-Japano-Koreanic language arrived with rice agriculture on the Korean peninsula around 1300 BC, is contradicted, first, by the absence of common rice vocabulary in Proto-Japano-Koreanic and second, by the early date proposed for the split between both families.

Although the agricultural vocabulary shared between Japonic and Koreanic is rather extensive with, for instance, a distinction between different terms for ‘field’, such as ‘field for cultivation’, ‘uncultivated field’ and ‘delimited plot for agriculture’, the languages do not have any rice vocabulary in common (Whitman, 2011; Robbeets, 2020). This observation indicates that the separation between both language families must predate the introduction of rice farming and should thus not be situated on the Korean peninsula, but rather in northeast China. The ancestral speech community must have been located to the north of the cultures on the Yellow River and the Shandong peninsula that were familiar with both millet and rice agriculture from at least the fourth millennium BC onwards.

The early date proposed for the separation between both families corroborates a split pre-dating the introduction of rice agriculture. Contrary to Kim and Park's (2020, p. 6) understanding that ‘While historical linguistics is interested in the timing and routes of language dispersal, it cannot directly address the temporality of these phenomena’, different linguistic dating techniques are used in the scholarly literature. For Proto-Japano-Koreanic, the different approaches converge on dating the break-up well before 1500 BC, to around the third millennium BC. Lexicostatistic dating estimates range from 4300 BC (Blažek & Schwarz, 2014, p. 88) to the fourth millennium BC (Starostin et al., 2003, p. 236) to the third millennium BC (Dybo & Korovina, 2019) to 2900 BC (Blažek & Schwarz, 2009); Bayesian methods infer a split at 1847 BC (Robbeets & Bouckaert, 2018) and cultural reconstruction (Robbeets et al., 2020) at 2600 BC.

Given the above linguistic indications, in our view the most parsimonious hypothesis is that Proto-Macro-Koreanic arrived on the Korean peninsula before Proto-Japanic, it was adopted by local hunter–gatherers speaking a language of Nivkh-like typology, it possessed agricultural vocabulary except for rice-related words, and it adopted rice terminology later in its history from incoming Japanic speakers.

## Conclusions

In this paper we re-examined the archaeolinguistic evidence used to associate Neolithic millet farming dispersals with Proto-Macro-Koreanic. Archaeological evidence that millet spread from west-central to southern Korea in association with Chulmun (comb-pattern) pottery, agricultural stone tools and textile technology supports a farming dispersal in the fourth millennium BC. While hunting, gathering and fishing retained an important role in Neolithic subsistence, millet cultivation probably formed the economic advantage which enabled this dispersal. A population increase around the time of the introduction of millet farming has been reported by Korean scholars. A subsequent apparent decrease in population after around 3000 BC is not evidence against a Middle Neolithic arrival of Proto-Macro-Koreanic. Instead, this decrease appears to be part of a broader Late Neolithic decline found in many regions of Eurasia. Plague (*Y. pestis*) was suggested as one possible cause of this apparently simultaneous population decline in Korea and Japan.

## Data Availability

All data used for this article can be found in the cited literature.
